# Descriptive Trends in Medicaid Antipsychotic Prescription Claims and Expenditures, 2016 – 2021

**DOI:** 10.1007/s11414-024-09889-0

**Published:** 2024-07-10

**Authors:** Nicole C. Giron, Hyesung Oh, Emily Rehmet, Theresa I. Shireman

**Affiliations:** 1 Department of Health Services, Policy & Practice, Brown University School of Public Health, Providence, RI USA; 2 UC Berkeley School of Law, Berkeley, CA USA; 3 Center for Gerontology and Healthcare Research, Brown University School of Public Health, Providence, RI USA

## Introduction

Medicaid covers nearly one-quarter of adults with mental illness in the United States [1], and antipsychotics have historically been a top drug class for Medicaid spending. In 2019, psychotherapeutic agents, which include antipsychotics and antidepressants, were the third most costly outpatient class for the program. Antipsychotic agents represented 9% of all Medicaid spending ($6.2 billion) and 10% of all Medicaid prescriptions (73.1 million claims) that year [2]. With the outsized role Medicaid plays in paying for behavioral health care and the growing number of beneficiaries in the program since the Affordable Care Act [3], it is important to monitor how and where resources are being devoted within the program.

Over the past two decades, several generic antipsychotic medications entered the market. After an originator drug product (i.e., brand-name medication) loses its patent protection, generic manufacturers may enter the market with typically lower priced, bioequivalent substitutes. Generic drug substitutes hold the promise of reducing pharmaceutical expenditures, while being just as effective as their originators [4, 5]. Prior work also has predicted that the introduction of generic antipsychotics should lead to substantial decreases in spending on brand-name medications within the Medicaid program [6]. Additionally, most state Medicaid programs require generic substitution unless a brand-name version is a medical necessity, and some implement policies such as prior authorizations aimed at reducing expenditures by increasing the use of preferred drugs, like generic versions of brand-name medications [7, 8]. Yet, some other studies have observed declines in antipsychotic prescriptions and use among certain Medicaid populations, despite the introduction of new antipsychotic medications [9, 10]. Examining recent national trends that can further inform behavioral health care in the Medicaid program is warranted.

This study sought to understand recent trends in Medicaid spending and prescribing patterns given the introduction of new antipsychotics and generic alternative to previously branded drugs. To examine these trends, this study provides a novel summary of longitudinal antipsychotic medication gross expenditure and prescription claim trends from 2016 to 2021 using data from the publicly available Centers for Medicare & Medicaid Services (CMS) Medicaid Spending by Drug Dashboard [11].

## Methods

### Data source

The authors extracted and analyzed the antipsychotic prescription and spending data from the CMS Medicaid Spending by Drug Dashboard. The Medicaid Dashboard captures data on claims and gross expenditures for Medicaid beneficiaries’ covered outpatient medication prescriptions [11] While the Medicaid Dashboard summarizes overall federal and state reimbursement for medications, it does not reflect state-specific spending, claims, or rebates. Nevertheless, this data provides the novel opportunity to generate trends summaries of the antipsychotic market within the Medicaid program.

All antipsychotic medications that had a product on the market at any point during the study period were included (2016 – 2021). Medication subclass (typical antipsychotics, which reflect the first generation of agents that primarily work through dopamine receptors, and atypical antipsychotics, which are second generation agents with fewer extra-pyramidal side effects as they work through serotonin receptors) [12], product type, and version (generic or brand-name) were distinguished by year. Products that shared the same active ingredients, combining dosages and routes of administration, were grouped. Use and spending across atypical antipsychotics that were newly introduced during the study period and/or represented less than 1% of the total Medicaid antipsychotic market across all years were collapsed. Use and spending across typical antipsychotics that represented less than 0.5% of the total Medicaid antipsychotic market across all years were also collapsed. The full list of included medications is available in Appendix Table 1.

### Analysis

Data were descriptively analyzed. Prescription claims and spending were calculated by subclass, version, product, and total for each year. Percentage change in total spending and claims during the study period was also calculated.

Next, new generic antipsychotic medications introduced since the early 2000s were identified (Appendix Table 2). These were all atypical antipsychotics. To understand the potential impact of generic introduction, the authors explored rates of generic substitution among each atypical medication (i.e., generic claims divided by the sum of generic and brand-name claims for a specific product). The overall generic substitution rate for atypical antipsychotics was also calculated.

Lastly, as a sensitivity analysis, how rebates may have impacted overall spending trends was examined. Actual rebates from pharmaceutical manufacturers are proprietary, so rebates were estimated by applying the base Medicaid rebate amount set by the Affordable Care Act (23.1% to brand-name and 13% to generic medications) [13].

Analyses were performed using Stata, version 17.0, between June 2022 and October 2023. STROBE guidelines for cross-sectional studies were incorporated in the development of this study [14]. This study used publicly available data and was exempt from the institutional review board (IRB).

## Study Results

### Overall trends at a glance

**Figure 1:**
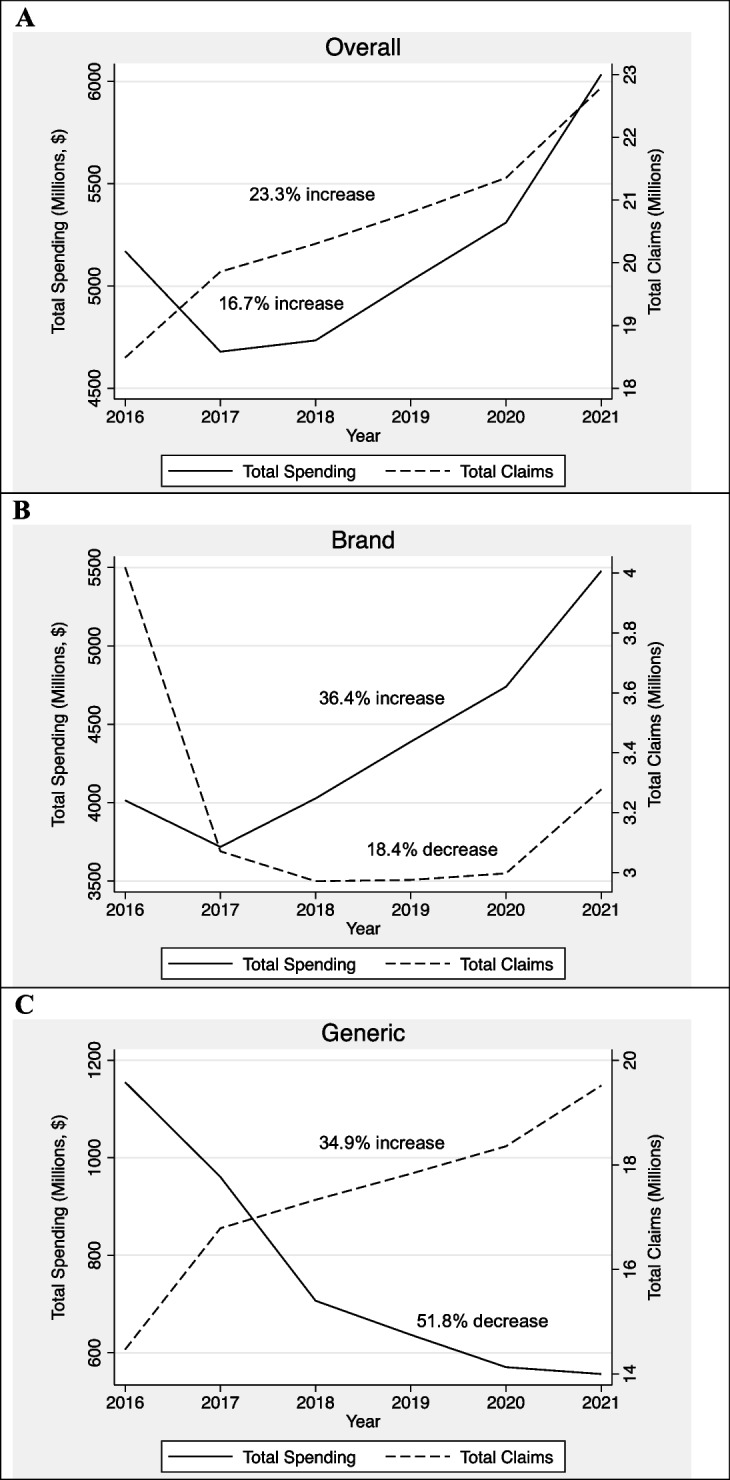
Prescription claims versus spending trends for all, generic, and brand-name antipsychotics, 2016 – 2021. Source: Authors’ analysis of the Centers for Medicare & Medicaid Services (CMS) Medicaid Spending by Drug Dashboard, 2016 – 2021

Both overall spending and prescription claims for antipsychotics increased between 2016 and 2021 (Fig. 1A), but brand-name medications drove gross spending increases despite representing a smaller proportion of total prescription claims (Fig. 1B). The opposite trend occurred among generic medications; claims for generics increased while their associated spending was halved during the same period (Fig. 1C). By 2021, brand-name medications represented 90.8% of all gross Medicaid spending on antipsychotic medications but only 14.4% of antipsychotic prescription claims.

### Prescription claim trends

The total number of prescription claims for antipsychotics increased by 23.3% from 18.5 million claims in 2016 to 22.8 million claims in 2021 (Table 1). Generic claims drove the bulk of this growth and increased by 34.9% from 14.5 million claims in 2016 to 19.5 million claims in 2021. Generics represented 85.6% of the total claims at the end of this period. Most of the growth in generic claims came from an increase in atypical claims, which increased by 37.7% during the period (compared to just 9.2% growth in typical generics). In 2021, generic atypical antipsychotics alone represented 78.8% of all antipsychotic claims.

**Table 1:** Prescription claims and spending for antipsychotics by subgroup and version, 2016 – 2021

	2016		2017		2018		2019		2020		2021		% change 2016 – 2021
	**Prescription claims (millions)**												
	*N*	%	*N*	%	*N*	%	*N*	%	*N*	%	*N*	%	
*Atypical antipsychotics*													
Generic	13.0	70.5	15.1	76.1	15.7	77.2	16.3	78.3	16.9	78.9	18.0	78.8	37.7
Brand	4.0	21.7	3.1	15.5	3.0	14.6	3.0	14.3	3.0	14.0	3.3	14.4	−18.4
Total	17.1	92.3	18.2	91.6	18.6	91.8	19.3	92.6	19.8	93.0	21.2	93.2	24.5
*Typical antipsychotics*													
Generic	1.4	7.7	1.7	8.4	1.7	8.2	1.5	7.4	1.5	7.0	1.6	6.8	9.2
Brand	0.0	0.0	0.0	0.0	0.0	0.0	0.0	0.0	0.0	0.0	0.0	0.0	−32.1
Total	1.4	7.7	1.7	8.4	1.7	8.2	1.5	7.4	1.5	7.0	1.6	6.8	9.1
*All antipsychotics*													
Generic	14.5	78.3	16.8	84.5	17.3	85.4	17.8	85.7	18.4	86.0	19.5	85.6	34.9
Brand	4.0	21.7	3.1	15.5	3.0	14.6	3.0	14.3	3.0	14.0	3.3	14.4	−18.4
Total	18.5		19.9		20.3		20.8		21.4		22.8		23.3
	**Prescription spending (millions)**												
	$	%	$	%	$	%	$	%	$	%	$	%	
*Atypical antipsychotics*													
Generic	1054.1	20.4	777.4	16.6	548.1	11.6	479.2	9.5	417.5	7.9	422.3	7.0	–59.9
Brand	4014.7	77.7	3718.0	79.5	4027.2	85.1	4388.8	87.3	4739.5	89.3	5477.2	90.8	36.4
Total	5068.8	98.0	4495.4	96.1	4575.3	96.6	4868.0	96.9	5157.0	97.1	5899.4	97.8	16.4
*Typical antipsychotics*													
Generic	101.0	2.0	183.5	3.9	158.7	3.4	157.8	3.1	153.1	2.9	134.2	2.2	32.8
Brand	0.3	0.0	0.2	0.0	0.1	0.0	0.1	0.0	0.1	0.0	0.1	0.0	–73.4
Total	101.3	2.0	183.7	3.9	158.8	3.4	157.9	3.1	153.1	2.9	134.3	2.2	32.6
*All antipsychotic*s													
Generic	1155.1	22.3	960.9	20.5	706.8	14.9	637.0	12.7	570.6	10.7	556.5	9.2	–51.8
Brand	4015.0	77.7	3718.2	79.5	4027.3	85.1	4389.0	87.3	4739.5	89.3	5477.2	90.8	36.4
Total	5170.1		4679.1		4734.1		5026.0		5310.1		6033.7		16.7

Source: Authors’ analysis of the Centers for Medicare & Medicaid Services (CMS) Medicaid Spending by Drug Dashboard, 2016 – 2021

Notes: Percentage (%) denotes the category’s proportional representation in the overall prescription count or spending amount within a given year. All numbers are rounded to the nearest tenth

While the number of claims for generic antipsychotics grew, the total number of brand-name claims declined during the same period. Total claims for brand-name medications decreased by 18.4% from 4.0 million claims in 2016 to 3.3 million claims in 2021. The decline in atypical brand-name claims almost exclusively drove the overall rate of decline during this period. Brand-name antipsychotics represented 21.7% of the total claims in 2016 but only 14.4% of the total claims in 2021.

### Spending trends

Total gross spending for antipsychotics increased by 16.7% from $5170.1 million in 2016 to $6033.7 million in 2021 (Table 1). Most Medicaid spending on antipsychotics during this period was for atypical prescriptions, which represented 97.8% of total antipsychotic spending by 2021.

However, when broken down by version, spending patterns did not follow prescription claim patterns. While claims for generic antipsychotics increased during the period, overall spending on generics decreased by 51.8% from $1155.1 million in 2016 to $556.5 million in 2021. Overall spending on brand-name antipsychotics increased by 36.4% from $4015.0 million in 2016 to $5477.2 million in 2021, despite a decrease in brand-name claims during the period. By 2021, overall gross spending on brand-name antipsychotics represented 90.8% of total spending on antipsychotics. Rebate estimates had little effect on overall spending trends (Appendix Table 3).

### Atypical antipsychotics prescriptions and spending

These trends were further explored by examining claims and spending by product, with a focus on atypical antipsychotics since these products drove most outpatient antipsychotic spending and prescribing between 2016 and 2021 (Table 2).

**Table 2:** Prescription claims and spending for atypical and typical antipsychotics, 2016 – 2021

	2016		2017		2018		2019		2020		2021		% change 2016-21
	**Prescription claims (millions)**												
**Antipsychotic classes/agents**	*N*	%	*N*	%	*N*	%	*N*	%	*N*	%	*N*	%	
*Atypical antipsychotics*													
Aripiprazole	3.3	17.7	3.5	17.5	3.7	18.1	4.0	19.1	4.3	20.1	4.8	22.5	45.8
Lurasidone	0.9	4.7	1.0	4.9	1.0	5.0	1.1	5.1	1.1	5.2	1.2	5.2	36.9
Olanzapine	1.9	10.3	2.0	10.2	2.1	10.3	2.2	10.4	2.3	10.6	2.5	11.6	29.4
Paliperidone	0.5	2.9	0.6	2.9	0.7	3.4	0.8	3.8	0.8	3.9	0.9	4.2	68.3
Quetiapine	5.1	27.4	5.6	28.0	5.7	28.1	5.8	27.6	5.9	27.6	6.2	29.3	23.0
Risperidone	3.7	20.3	3.8	19.0	3.6	18.0	3.6	17.3	3.4	16.0	3.4	16.2	−8.1
Ziprasidone	0.8	4.5	0.8	4.1	0.8	3.7	0.7	3.4	0.7	3.1	0.7	3.1	−20.1
Other	0.8	4.5	1.0	4.9	1.1	5.3	1.2	5.9	1.4	6.4	1.6	7.4	88.4
Total	17.1	92.3	18.2	91.6	18.6	91.8	19.3	92.6	19.8	93.0	21.2	93.2	24.5
*Typical antipsychotics*													
Chlorpromazine HCl	0.1	0.4	0.2	1.2	0.2	1.2	0.2	1.1	0.2	1.1	0.2	1.0	203.3
Fluphenazine	0.2	0.9	0.2	0.9	0.2	0.9	0.2	0.9	0.2	0.8	0.2	0.7	−3.2
Haloperidol	1.0	5.3	1.0	5.0	1.0	4.8	0.9	4.4	0.9	4.3	1.0	4.3	−0.6
Perphenazine	0.1	0.4	0.2	0.8	0.2	0.7	0.1	0.7	0.1	0.6	0.1	0.6	62.5
Other	0.1	0.7	0.1	0.6	0.1	0.6	0.1	0.3	0.1	0.3	0.1	0.3	−48.6
Total	1.4	7.7	1.7	8.4	1.7	8.2	1.5	7.4	1.5	7.0	1.6	6.8	9.1
Sum subclasses	18.5		19.9		20.3		20.8		21.4		22.8		23.3
	**Prescription spending (millions)**												
	$	%	$	%	$	%	$	%	$	%	$	%	
*Atypical antipsychotics*													
Aripiprazole	1983.5	38.4	1253.1	26.8	1121.0	23.7	997.3	19.8	855.3	16.1	976.9	16.2	−50.7
Lurasidone	871.6	16.9	1101.9	23.5	1246.0	26.3	1339.7	26.7	1464.9	27.6	1640.9	27.2	88.3
Olanzapine	160.3	3.1	66.9	1.4	55.9	1.2	64.2	1.3	49.0	0.9	52.1	0.9	−67.5
Paliperidone	863.7	16.7	1039.0	22.2	1216.2	25.7	1436.1	28.6	1542.7	29.1	1738.0	28.8	101.2
Quetiapine	621.3	12.0	365.8	7.8	172.5	3.6	105.3	2.1	94.3	1.8	96.3	1.6	−84.5
Risperidone	204.6	4.0	190.3	4.1	170.8	3.6	166.9	3.3	159.6	3.0	157.2	2.6	−23.2
Ziprasidone	78.8	1.5	48.2	1.0	35.8	0.8	28.1	0.6	24.0	0.5	25.7	0.4	−67.3
Other	285.1	5.5	430.2	9.2	557.0	11.8	730.5	14.5	967.2	18.2	1212.3	20.1	325.2
Total	5068.8	98.0	4495.4	96.1	4575.3	96.6	4868.0	96.9	5157.0	97.1	5899.4	97.8	16.4
*Typical antipsychotics*													
Chlorpromazine HCl	23.9	0.5	92.4	2.0	81.9	1.7	70.1	1.4	62.4	1.2	52.3	0.9	118.6
Fluphenazine	18.3	0.4	28.8	0.6	23.6	0.5	37.8	0.8	40.9	0.8	31.9	0.5	74.6
Haloperidol	44.6	0.9	42.5	0.9	38.1	0.8	40.1	0.8	41.3	0.8	41.3	0.7	−7.4
Perphenazine	7.1	0.1	12.4	0.3	8.8	0.2	6.6	0.1	5.2	0.1	5.2	0.1	−26.5
Other	7.4	0.1	7.6	0.2	6.4	0.1	3.3	0.1	3.4	0.1	3.6	0.1	−51.3
Total	101.3	2.0	183.7	3.9	158.8	3.4	157.9	3.1	153.1	2.9	134.3	2.2	32.6
Sum subclasses	5170.1		4679.1		4734.1		5,026.0		5310.1		6033.7		16.7

Source: Authors’ analysis of the Centers for Medicare & Medicaid Services (CMS) Medicaid Spending by Drug Dashboard, 2016 – 2021

Notes: Percentage (%) denotes the category’s proportional representation in the overall prescription count or spending amount within a given year. All numbers are rounded to the nearest tenth. Medications included in “Other Atypical Antipsychotics:” asenapine, brexpiprazole, cariprazine, clozapine, iloperidone, lumateperone, pimavanserin. Medications included in “Other Typical Antipsychotics:” loxapine, molindone, thioridazine, thiothixene, trifluoperazine

Quetiapine (29.3%) and aripiprazole (22.5%) contributed the largest proportion of all claims among atypical medications with a generic version in 2021. Nearly two-thirds of total spending in 2021 was attributed to paliperidone (28.8%). Risperidone (8.1% decrease in claims, from 3.7 million to 3.4 million) and ziprasidone (20.1% decrease in claims, from 0.8 million to 0.7 million) were the only atypical antipsychotics with a generic version on the market to experience decreases in overall prescribing between 2016 and 2021. Prescribing for all other atypical medications with a generic version increased during the period. Paliperidone (101.2% increase in spending, from $863.7 million to $1738.0 million) was the only atypical antipsychotic with a generic version during the full period to experience an increase in spending. Spending on all other atypical medications with a generic version decreased during this period.

Notably, atypical antipsychotics with only brand-name versions on the market appeared to have had a sizeable impact on the market. In 2021, lurasidone represented 27.2% of Medicaid spending in 2021 and had the second-largest relative growth over the period among individual atypical products (88.3% increase in spending, from $871.6 million to $1640.9 million). The largest relative growth in spending over time came from the collection of the medications included in the “Other atypical antipsychotics” group (i.e., asenapine, brexpiprazole, cariprazine, clozapine, iloperidone, lumateperone, pimavanserin). Most of the medications in this group were atypical medications with only brand-name versions on the market during all or most of the study period. The only exception was clozapine, which had a generic version on the market during the full period but was included in this group because it represented a small proportion of total claims. While claims among this “other” group grew by 88.4% during the period, from 0.8 million in 2016 to 1.6 million in 2021, spending associated with these medications increased by 325.2% in the same period, from $285.1 million to $1212.3 million.

### Generic substitution among atypical antipsychotics

Among atypical antipsychotics with both a generic and brand-name version on the market, the overall rate of generic substitution of brand-name medications increased from 81.8% in 2016 to 93.7% in 2021 (Table 3). Generic substitution rates varied by product. In 2016, the second year of their generic availability, generic paliperidone accounted for 16.2% of all paliperidone claims while generic aripiprazole accounted for 62.1% of all aripiprazole claims. By 2021, generic paliperidone grew to account for only 30.2% of all paliperidone claims. All other atypical antipsychotics that also had a generic version on the market every year between 2016 and 2021 had reached generic substitution rates greater than 90% by the end of the period. Notably, asenapine was the only product to have a generic version introduced during this period (in 2020), and claims for its generic version represented over half of all asenapine claims within one year of its introduction.

**Table 3:** Generic substitution rates for atypical antipsychotics, 2016 – 2021

Atypical product	2016	2017	2018	2019	2020	2021
Overall	81.8%	89.9%	91.4%	92.7%	93.5%	93.7%
Clozapine	95.1%	98.2%	98.9%	99.1%	99.5%	99.6%
Olanzapine	93.6%	99.0%	99.3%	99.3%	99.5%	99.6%
Quetiapine	83.6%	95.4%	98.2%	99.5%	99.7%	99.8%
Risperidone	95.3%	95.9%	96.3%	96.6%	96.9%	97.1%
Ziprasidone	94.5%	97.8%	97.7%	97.5%	97.7%	98.2%
Aripiprazole	62.1%	78.0%	81.5%	86.7%	91.7%	91.8%
Paliperidone	16.2%	18.0%	24.2%	28.4%	31.5%	30.2%
Asenapine	—	—	—	—	0.2%	52.7%
Brexpiprazole	—	—	—	—	—	—
Cariprazine	—	—	—	—	—	—
Iloperidone	—	—	—	—	—	—
Lumateperone	—	—	—	—	—	—
Lurasidone	—	—	—	—	—	—
Pimavanserin	—	—	—	—	—	—

Source: Authors’ analysis of the Centers for Medicare & Medicaid Services (CMS) Medicaid Spending by Drug Dashboard, 2016 – 2021

Notes: Percentage (%) denotes each product’s generic proportional representation in the product’s overall prescription market share. Products without data represent those without a generic version on the market. The calculation for the overall rate includes only products with both a generic and brand-name version on the market in the given year. All numbers are rounded to the nearest tenth

## Discussion

This study finds that the introduction of generics may have helped to expand access to antipsychotic medications; when available, generic antipsychotics appeared to have been prescribed at higher rates than brand-name medications at a lower cost during this period. However, brand-name medications drove the bulk of gross Medicaid spending on antipsychotics despite their relatively lower use, which reflects patterns observed in the overall Medicaid drug market [7]. In 2021, the most recent year for which data was available at the time this study’s analysis was conducted, the overwhelming majority of gross Medicaid spending was devoted to reimbursements for brand-name medications.

Atypical antipsychotics drove outpatient prescriptions and gross spending during the period. Among atypical antipsychotics, the most expensive medications appear to be those without a generic option available. Notably, most atypical antipsychotics that saw increases in spending were those without generic options during some or all of the period: lurasidone (Latuda), asenapine (Saphris, Secuado), brexpiprazole (Rexulti), iloperidone (Fanpt), lumateperone (Caplyta), and pimavanserin (Nuplazid). Paliperidone, the only atypical product representing a larger proportion of claims, with a generic version on the market during the full period, and with associated increases in spending, was also the product with the lowest generic substitution rate over time.

This study has some limitations. First, this study does not examine the health outcomes associated with antipsychotic use. Rising antipsychotic use and expense may be offset with better health outcomes, thereby improving the quality and quantity of lives for those who use them. Second, the Medicaid dashboard data only includes information on overall outpatient prescriptions and spending. While study results cannot be generalized to the inpatient setting, where trends may be different, most adults with mental illness in the United States receive prescription medication and/or outpatient mental health services [15]. Additionally, the dashboard did not include information on the unique number of beneficiaries served. Thus, our analyses could not account for total Medicaid beneficiaries represented in the data, which may be a factor impacting observed trends. Lastly, rebate information is not available in the Medicaid dashboard, which means the dollar values reported from the CMS website are undoubtedly higher than what states’ Medicaid programs pay. The authors applied the federally mandated, base rebate amounts (23% brands/13% generics) and show those adjusted expenditures in Appendix Table 3. These estimates are likely to remain high. Rebate calculations include an inflationary component in addition to the base rate to account for increases in drug pricing over time, and states and managed care plans negotiate with manufacturers for supplemental rebates [13]. The impact of these additional rebate components can be especially substantial on older brand-name drugs, which can be cheaper than newer or generic counterparts after accounting for the full rebate amount. Additionally, Medicaid beneficiaries may receive outpatient medications via a providers’ 340B Drug Pricing Program, which allows participating providers to procure drugs at a reduced rate to serve safety net populations. While 340B ceiling prices match Medicaid net rebate prices, pharmaceutical companies can offer additional discounts to 340B providers, which may impact overall Medicaid spending and are not captured in this analysis [13]. Nevertheless, this study’s longitudinal exploration of gross Medicaid spending provides helps to illuminate informative trends for a vital and impactful area of national mental health care.

These research findings highlight important considerations about spending and prescribing of antipsychotic drugs in the Medicaid market. First, these results highlight potential market inefficiencies in a public insurance market. Brand-name pharmaceutical companies have great incentive to maintain or increase high prices for originator drug products and have many avenues to do so [16]. These tactics may have contributed to generic antipsychotic substitution delay from 2016 to 2021. For example, pharmaceutical companies often block generic entry to maintain market dominance for brand-name products through the practice of evergreening, where they extend their brand products’ exclusivity period and prevent generic substitution through patent extensions using litigation and reformulations [17–20]. A patent infringement lawsuit regarding brand-name Latuda delayed generic antipsychotic entry into the market to 2023 [21]. Companies may also engage product hopping, one form of evergreening where patents are extended by the creation of new, often minor, product formulations or new delivery modalities, which can successfully delay generic market entries [18]. In anticipation of generic displacement of Invega and Invega Sustenna, Janssen pharmaceuticals introduced a paliperidone reformulation product (Invega Trinza) in 2015 claiming it would improve drug compliance [22].

Second, there may be important clinical, or financial reasons that prevent switching from brand-name to generic drugs. Clinically, maintaining high levels of adherence among patients is of utmost importance for improved health outcomes. Switching patients’ medications can lead to new side effects, changes in delivery modalities, or increases in treatment costs due to resulting adverse health outcomes, all of which may disrupt adherence [23]. Additionally, there may be existing structural and resource barriers that prevent marginalized communities covered by Medicaid from regularly accessing mental health care and receiving prescriptions for new medications, including newly introduced generics [24]. Financially, state Medicaid agencies and providers may be choosing to continue prescribing brand-name versions even when generics exist because existing brand-name versions may be cheaper after accounting for full rebate amounts [13]. In these situations, switching to generic substitutes or even new brand-name drugs may not be the best clinical or financial decision for Medicaid beneficiaries.

Lastly, existing state policies may shape uptake of generic medications. Most states have policies in place that modulate pharmaceutical prescription patterns, such as prior authorizations, step therapy, or monthly caps. These policies may limit providers’ ability to prescribe new medications if they are more expensive than existing options. While these policies are aimed at reducing expenditures while increasing the use of preferred medications, earlier work on prior authorizations has found that they can instead reduce the use of targeted medications after implementation [8, 9]. It is important to further understand the role that mental health care access, costs, and existing policy play in driving existing antipsychotic prescription and spending trends.

## Implications for Behavioral Health

These results suggest that brand-name antipsychotics have been able to generate substantial revenues even in the face of increasing generic penetration. This has had a significant impact on state and federal Medicaid spending and highlights the need for additional research and policy into pharmaceutical market sustainability within the antipsychotic space.

Importantly, state Medicaid programs must cover essentially all drugs approved by the Food & Drug Administration but are currently limited in their ability to directly address pricing issues. This has resulted in utilization-focused strategies to curb expenditures, such as narrowing coverage criteria and setting prescription limits, which may have minimal effects on total expenditures [25]. State Medicaid programs, policymakers, and researchers should revisit the definition of medical necessity in Medicaid pharmaceutical policies to ensure clear provider guidelines for prescriptions and pursue policies that directly target the pricing of branded pharmaceuticals. It is also important for further research to examine provider prescribing patterns after the market introduction of generic counterparts, as well as how state policies can impact these patterns. Further examination into these national trends may inform policy efforts to balance cost savings goals with clinical decision making that focuses on promoting adherence and the benefits of existing medications.

## Supplementary Information

ESM 1 (DOCX 30 kb)
