# Supplementary material for: Descriptive Trends in Medicaid Antipsychotic Prescription Claims and Expenditures, 2016 – 2021
Source: J Behav Health Serv Res. 2024 Jul 10;51(4):516–28. doi: 10.1007/s11414-024-09889-0 (PMC11445305; doi:10.1007/s11414-024-09889-0)
Supplement: Supplementary file 1 — (DOCX 30 kb) [file 11414_2024_9889_MOESM1_ESM.docx]

**Appendix Exhibits**

| Appendix Table 1. List of Antipsychotic Medications | | |  |  |
| --- | --- | --- | --- | --- |
|  | **Brand Name (if available)** | **Generic Name** | **Product** | **Use** |
| Typical Antipsychotics | - | Chlorpromazine HCl | Chlorpromazine | Bipolar disorder, hyperactivity, schizophrenia |
|  | - | Fluphenazine Decanoate | Fluphenazine | Psychotic disorders |
|  | - | Fluphenazine HCl | Fluphenazine |  |
|  | - | Haloperidol | Haloperidol | Hyperactivity, Tourette syndrome, schizophrenia |
|  | - | Haloperidol Decanoate 100 | Haloperidol |  |
|  | Haldol Decanoate 100 | Haloperidol Decanoate 100 | Haloperidol |  |
|  | - | Haloperidol Decanoate 50 | Haloperidol |  |
|  | Haldol Decanoate 50 | Haloperidol Decanoate 50 | Haloperidol |  |
|  | Haldol | Haloperidol Lactate | Haloperidol |  |
|  | - | Haloperidol Lactate | Haloperidol |  |
|  | - | Loxapine Succinate | Loxapine | Schizophrenia |
|  | - | Molindone HCl | Molindone | Schizophrenia |
|  | - | Perphenazine | Perphenazine | Schizophrenia |
|  | - | Perphenazine Amitriptyline HCl | Perphenazine |  |
|  | - | Thioridazine HCl | Thioridazine | Schizophrenia |
|  | - | Thiothixene | Thiothixene |  |
|  | - | Trifluoperazine HCl | Trifluoperazine | Schizophrenia |
| Atypical Antipsychotics | Abilify Mycite | Aripiprazole | Aripiprazole | Acute agitation, bipolar disorder, depression, irritability associated with autistic disorder, Tourette’s syndrome, and schizophrenia |
|  | Abilify | Aripiprazole | Aripiprazole |  |
|  | - | Aripiprazole | Aripiprazole |  |
|  | Abilify Maintena | Aripiprazole | Aripiprazole |  |
|  | Aristada | Aripiprazole Lauroxil | Aripiprazole |  |
|  | Aristada Initio | Aripiprazole Lauroxil Submicr. | Aripiprazole |  |
|  | - | Aripiprazole ODT | Aripiprazole |  |
|  | Secuado | Asenapine | Asenapine | Acute mania associated with bipolar disorder, and schizophrenia |
|  | - | Asenapine Maleate | Asenapine |  |
|  | Saphris | Asenapine Maleate | Asenapine |  |
|  | Rexulti | Brexpiprazole | Brexpiprazole | Depression, agitation associated with dementia due to Alzheimer’s disease, schizophrenia |
|  | Vraylar | Cariprazine HCl | Cariprazine | Bipolar disorder, schizophrenia |
|  | Clozaril | Clozapine | Clozapine | Schizophrenia |
|  | - | Clozapine | Clozapine |  |
|  | Versacloz | Clozapine | Clozapine |  |
|  | Fazaclo | Clozapine | Clozapine |  |
|  | - | Clozapine ODT | Clozapine |  |
|  | Fanapt | Iloperidone | Iloperidone | Bipolar disorder, schizophrenia |
|  | Caplyta | Lumateperone Tosylate | Lumateperone | Schizophrenia |
|  | Latuda | Lurasidone HCl | Lurasidone | Depression associated with bipolar disorder, schizophrenia |
|  | - | Olanzapine | Olanzapine | Bipolar disorder, treatment resistant depression, schizophrenia |
|  | Zyprexa | Olanzapine | Olanzapine |  |
|  | Zyprexa Zydis | Olanzapine | Olanzapine |  |
|  | Symbyax | Olanzapine Fluoxetine HCl | Olanzapine |  |
|  | - | Olanzapine Fluoxetine HCl | Olanzapine |  |
|  | - | Olanzapine ODT | Olanzapine |  |
|  | Zyprexa Relprevv | Olanzapine Pamoate | Olanzapine |  |
|  | Lybalvi | Olanzapine Samidorphan Malate | Olanzapine |  |
|  | Invega | Paliperidone | Paliperidone | Schizophrenia |
|  | - | Paliperidone ER | Paliperidone |  |
|  | Invega Hafyera | Paliperidone Palmitate | Paliperidone |  |
|  | Invega Sustenna | Paliperidone Palmitate | Paliperidone |  |
|  | Invega Trinza | Paliperidone Palmitate | Paliperidone |  |
|  | Nuplazid | Pimavanserin Tartrate | Pimavanserin | Parkinson’s disease  psychosis |
|  | Seroquel XR | Quetiapine Fumarate | Quetiapine | Bipolar disorder, depression, and schizophrenia |
|  | Seroquel | Quetiapine Fumarate | Quetiapine |  |
|  | - | Quetiapine Fumarate | Quetiapine |  |
|  | - | Quetiapine Fumarate ER | Quetiapine |  |
|  | - | Risperidone | Risperidone | Bipolar disorder, irritability associated with autism, schizophrenia |
|  | Perseris | Risperidone | Risperidone |  |
|  | Risperdal | Risperidone | Risperidone |  |
|  | Risperdal Consta | Risperidone Microspheres | Risperidone |  |
|  | - | Risperidone ODT | Risperidone |  |
|  | - | Ziprasidone HCl | Ziprasidone | Bipolar disorder, acute agitation due to schizophrenia, and schizophrenia |
|  | Geodon | Ziprasidone HCl | Ziprasidone |  |
|  | - | Ziprasidone Mesylate | Ziprasidone |  |
|  | Geodon Mesylate | Ziprasidone Mesylate | Ziprasidone |  |

**SOURCE** Authors’ analysis of the Centers for Medicare & Medicaid Services (CMS) Medicaid Spending by Drug Dashboard, 2016 – 2021 and analysis of announcements and Federal Drug Administration (FDA) records available online.

**Appendix Table 2. Antipsychotic and generic entry year, 2008-2021**

| **Antipsychotic Class/Agent** | **Brand Name(s)** | **Generic Name(s)** | **Generic Entry Year** |
| --- | --- | --- | --- |
| Risperidone | Risperdal | Risperidone | 2008 |
| Olanzapine | Zyprexa | Olanzapine | 2011 |
| Quetiapine | Seroquel | Quetiapine | 2012 |
| Aripiprazole | Abilify | Aripiprazole | 2015 |
| Paliperidone | Invega | Paliperidone | 2015 |
| Quetiapine ER | Seroquel XR | Quetiapine Fumarate ER | 2017 |
| Ziprasidone | Geodon (Injectible) | Ziprasidone Mesylate | 2020 |
| Asenapine | Saphris, Secuado | Asenapine | 2020 |

**SOURCE** Authors’ analysis of announcements and records available online.

**Appendix Table 3.** Prescription spending with estimated rebates for antipsychotics by subgroup and version, 2016 – 2021

|  | **2016** | | **2017** | | **2018** | | **2019** | | **2020** | | **2021** | | **% change 2016-21** |
| --- | --- | --- | --- | --- | --- | --- | --- | --- | --- | --- | --- | --- | --- |
| **Prescription spending with estimated rebates (millions)** | | | | | | | | | | | | |  |
|  | **$** | **%** | **$** | **%** | **$** | **%** | **$** | **%** | **$** | **%** | **$** | **%** |  |
| Atypical Antipsychotics (Rebate) | | |  |  |  |  |  |  |  |  |  |  |  |
| Generic | 917.1 | 22.4 | 676.4 | 18.3 | 476.9 | 12.8 | 416.9 | 10.6 | 363.2 | 8.8 | 367.4 | 7.8 | -59.9 |
| Brand | 3087.3 | 75.4 | 2859.1 | 77.4 | 3096.9 | 83.4 | 3375.0 | 85.9 | 3644.7 | 88.0 | 4211.9 | 89.7 | 36.4 |
| **Total** | 4004.4 | 97.8 | 3535.5 | 95.7 | 3573.8 | 96.3 | 3791.9 | 96.5 | 4007.9 | 96.8 | 4579.3 |  | 14.4 |
|  |  |  |  |  |  |  |  |  |  |  |  |  |  |
| Typical Antipsychotics (Rebate) | | |  |  |  |  |  |  |  |  |  |  |  |
| Generic | 87.9 | 2.1 | 159.6 | 4.3 | 138.0 | 3.7 | 137.3 | 3.5 | 133.2 | 3.2 | 116.8 | 2.5 | 32.8 |
| Brand | 0.2 | 0.0 | 0.2 | 0.0 | 0.1 | 0.0 | 0.1 | 0.0 | 0.1 | 0.0 | 0.1 | 0.0 | -73.4 |
| **Total** | 88.1 | 2.2 | 159.8 | 4.3 | 138.1 | 3.7 | 137.4 | 3.5 | 133.2 | 3.2 | 116.8 | 2.5 | 32.6 |
|  |  |  |  |  |  |  |  |  |  |  |  |  |  |
| All Antipsychotics (Rebate) | | |  |  |  |  |  |  |  |  |  |  |  |
| Generic | 1005.0 | 24.6 | 836.0 | 22.6 | 614.9 | 16.6 | 554.2 | 14.1 | 496.4 | 12.0 | 484.1 | 10.3 | -51.8 |
| Brand | 3087.5 | 75.4 | 2859.3 | 77.4 | 3097.0 | 83.4 | 3375.1 | 85.9 | 3644.7 | 88.0 | 4212.0 | 89.7 | 36.4 |
| **Total** | **4092.5** |  | **3695.3** |  | **3711.9** |  | **3929.3** |  | **4141.1** |  | **4696.1** |  | **14.8** |

**SOURCE** Authors’ analysis of the Centers for Medicare & Medicaid Services (CMS) Medicaid Spending by Drug Dashboard, 2016 - 2021.

**NOTES** Percentage (%) denotes the category's proportional representation in the overall prescription count or spending amount within a given year.
